# Who knew? The misleading specificity of “double-blind” and what to do about it

**DOI:** 10.1186/s13063-020-04607-5

**Published:** 2020-08-05

**Authors:** Thomas A. Lang, Donna F. Stroup

**Affiliations:** 1 West China Hospital/Sichuan Medical School Publishing Group, Kirkland, WA USA; 2 Data for Solutions, Inc., Decatur, GA USA

**Keywords:** Random assignment, Allocation concealment, Blinding, Randomized trials, Surveillance bias, Expectation bias, Ascertainment bias, Trial reporting

## Abstract

**Background:**

In randomized trials, the term “double-blind” (and its derivatives, single- and triple-blind, fully blind, and partially blind or masked) has no standard or widely accepted definition. Agreement about which groups are blinded is poor, and authors using these terms often do not identify which groups were blinded, despite specific reporting guidelines to the contrary. Nevertheless, many readers assume—incorrectly—that they know which groups are blinded. Thus, the term is ambiguous at best, misleading at worst, and, in either case, interferes with the accurate reporting, interpretation, and evaluation of randomized trials. The problems with the terms have been thoroughly documented in the literature, and many authors have recommended that they be abandoned.

**Proposal:**

We and our co-signers suggest eliminating the use of adjectives that modify “blinding” in randomized trials; a trial would be described as either blinded or unblinded. We also propose that authors report in a standard table which groups or individuals were blinded, what they were blinded to, how blinding was implemented, and whether blinding was maintained. Individuals with dual responsibilities, such as caregiving and data collecting, would also be identified. If blinding was compromised, authors should describe the potential implications of the loss of blinding on interpreting the results.

**Conclusion:**

“Double blind” and its derivatives are terms with little to recommend their continued use. Eliminating the use of adjectives that impart a false specificity to the term would reduce misinterpretations, and recommending that authors report who was blinded to what and how in a standard table would require them to be specific about which groups and individuals were blinded.

## Background: problems with the term “double-blind”

The single biggest problem in communication is the illusion that it has taken place. George Bernard Shaw

In reports of randomized trials, the use of the term “double-blind” and its derivatives (single- triple-blind, fully blind, and partially blind or masked) is commonly understood to indicate that two groups participating in the trial are kept unaware of which participants are receiving the experimental intervention and which are receiving the control intervention [1–6].

Despite its long and widespread use, however, the term has several problems.

### It is ambiguous

Agreement about which groups are blinded in a double-blind trial is poor [1–16]. For example, in one study, 91 physicians reported 17 unique combinations of groups (often more than two) that they believed were blinded in a double-blind trial (Table 1), and 25 textbooks contained 9 unique combinations [1]. Another study of 25 “double-blind trials” published in 16 leading journals identified 5 different combinations of participants, assessors, caregivers, and statisticians as being blinded [14]. Identifying groups in general terms (e.g., investigators, caregivers) is also ambiguous [4], especially when individuals have dual roles, such as collecting data and assessing outcomes [2, 4–6].

**Table 1:**
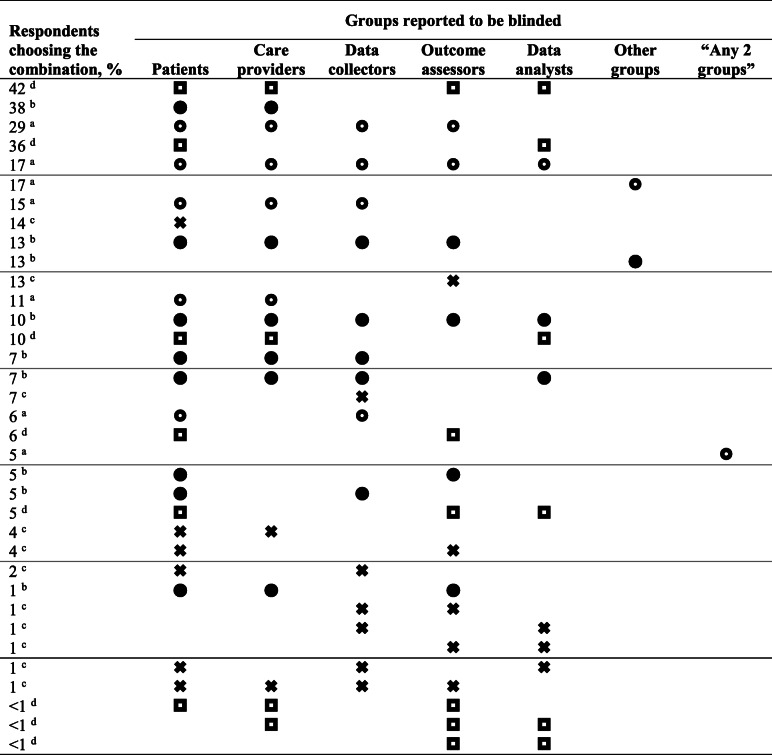
Groups reported to be blinded in a double-blind trial. Data are from 2 studies of () 130^a^ and () 91^b^ physicians and from 2 studies of () 83^c^ and () 194^d^ published randomized trials described as “double-blinded.” Combinations of 3 or more groups were often included in the definition of double-blind

^a^Data are from Table 5 in Haahr MT, Hróbjartsson A. Who is blinded in randomized clinical trials? A study of 200 trials and a survey of authors. *Clin Trials* 2006;3(4):360-5

^b^Data are from the table in Devereaux PJ, Manns BJ, Ghali WA, et al. Physician interpretations and textbook definitions of blinding terminology in randomized controlled trials. *JAMA*. 2001;285:2000-3. Of 17 unique group combinations, 9 are shown. Presumably, the remaining 8 combinations included “other groups” thought to be blinded (e.g., laboratory technicians, pharmacists)

^c^In this study of 83 “double-blind” trials, 49% (41) did not indicate which groups were blinded. Data are from Table 2 in Montori VM, Bhandari M, Devereaux PJ, et al. In the dark: the reporting of blinding status in randomized controlled trials. *J Clin Epidemiol* 2002;55:787-790

^d^Data are from Table 1 in Viergever RF, Ghersi D. Information on blinding in registered records of clinical trials. Trials 2012 Nov 15;13:210

### It is often uninformative

Even when using the term in an article, many authors do not identify which groups were blinded or how blinding was implemented [1–6, 9, 11, 12, 14, 16, 17]. Among 83 published trials reported as being double-blind, 41 did not identify any group as being blinded [9]. Without this information, “readers should remain skeptical about [blinding’s] effect on bias reduction.” [2].

### It can be misleading

Many readers assume—incorrectly—that they know which groups are blinded in a double-blind trial (Table 1) [2–5, 11, 15, 16]. Unfortunately, grossly inadequate reporting allows this assumption to go unchallenged when the article is read. (However, several studies have found that many published trials do not include the details of blinding, even when blinding was adequately implemented [4].) In 88 (70%) of 126 registered anesthesia trials, the groups or individuals reported to be blinded in the published results differed from those listed in the corresponding protocols [16].

### It is inadequate

The suggestion to establish explicit definitions for the term [7, 18] is complicated by the fact that several groups or individuals can be blinded. Limiting “double-blind” to trials in which only 2 specific groups are blinded leaves other combinations without an equivalent term.

### It is often confused with allocation concealment

In randomized trials, the allocation schedule (the list indicating the group to which the next participant will be assigned, in random order) has to be kept secret to prevent group assignment from being manipulated. That is, allocation concealment minimizes selection bias *before* participants have been assigned to experimental groups, whereas blinding minimizes surveillance, expectation, and ascertainment bias *after* group assignment. Many readers are not aware of this difference [2, 5, 6, 8, 12, 13, 15, 18–20], perhaps because the terms “allocation” and “blinding” indicate neither the similarities nor the differences between the concepts.

### It is often mistakenly believed to be required in a randomized trial and to be essential to the trial’s validity [1, 2, 5, 11, 13, 15, 16, 19–21]

“A randomised trial can be methodologically sound and not be double blind or, conversely, double blind and not methodologically sound.” [2]. Said another way, “Let us examine the placebo somewhat more critically, however, since it and ‘double blind’ have reached the status of fetishes in our thinking and literature. The Automatic Aura of Respectability, Infallibility, and Scientific Savoir-faire which they possess for many can be easily shown to be undeserved in certain circumstances.” [21].

### In some situations, it can be confused with the condition of being without sight [2, 5, 12, 20, 22, 23]

Some authors prefer “masking” to “blinding,” although the meaning of either term in a clinical trial may not be readily apparent to nonnative English speakers [18, 22]. Further, some authors use the terms interchangeably [5–7, 10–12, 15, 18, 24, 25], others insist that only masking be used [17, 20, 23], and still others insist that only blinding be used [2, 5, 22]. In addition, masking is sometimes used to describe *how* treatments are made indistinguishable [18, 19, 25, 26], whereas blinding usually indicates *which groups* are unaware of treatment assignment [1–6]. Finally, searching the literature for “blinded,” “partially blind,” or “fully blind” randomized trials also identifies dozens of unwanted citations to the condition of being without sight.

### It is unrealistic

The problem with trying to identify in a single term the groups who are blinded in a trial is that the number of pairs is potentially large. The literature identifies 11 groups or individuals who could be blinded: participants, care providers, data collectors and managers, trial managers, pharmacists [27], laboratory technicians [1], outcome assessors (who collect data on outcomes), outcome adjudicators (who confirm that an outcome meets established criteria), statisticians [2, 4, 6, 11–13], and sometimes even members of data monitoring and safety committees [1, 3, 4, 6, 11, 17] and manuscript writers [3, 6, 11, 16, 17]. These 11 groups can form 55 unique pairs. Even limiting the possibilities to 5 groups commonly recommended for blinding [15, 28]—participants, care providers, data collectors, outcome assessors, and statisticians—leaves 10 possible combinations.

## Proposed solutions

As near as we can tell, despite the above problems and several calls to abandon the term [1, 5, 6, 9, 11, 12, 16, 28], only one substitute for double-blinding has been proposed in the literature: “subject- and assessor-blind” [29]. Aside from being somewhat awkward, the term assumes that double-blinding applies only to subjects and assessors, which, although reasonable, is not uniformly accepted.

The terms “fully blinded” or “partially blinded” do appear in the literature, but not as substitutes for substitutes for double-blinding or single-blinding [27]. Although both are used in randomized trials, they involve randomly assigning treatments, not groups, and can be applied to subsets of individuals within groups. For example, participants who could receive either an active drug or a placebo would be “fully blinded,” whereas participants who know they are receiving an active drug but not which one, would be “partially blinded.”

We considered blinding “assignment concealment [24]” because it accurately indicates that group assignment is what is hidden. It does not imply which groups are involved and has no history of doing so. It also eliminates the blinding-masking controversy and is not associated with other, less-technical meanings. Further, the relationship between blinding and “allocation concealment” is not apparent, but allocation concealment and assignment concealment are two sides of the same coin: they clearly indicate that two different components of the trial are concealed: the allocation schedule and group assignment, one term indicating group concealment before assignment and one after.

However, assignment concealment does not work well as a label. We concluded that “a concealed assignment trial” was unlikely to replace “a blinded trial.” Likewise, its use can be awkward: “group assignment was concealed from participants” was unlikely to replace “participants were blinded to treatment.” Further, as noted above, for better or worse, the mere use of the term “blinding” is widely considered to indicate study quality, and we concluded that authors would be unwilling to give up using this prized and familiar term. Finally, many people believed that “concealment” should be reserved for, or would be confused with, allocation concealment.

## Proposal

The term “blinding” is so firmly established that a simple substitute term, even if we could find one, is unlikely to be acceptable. Instead, we propose two changes in reporting trials described as blinded.

Our first proposal is to eliminate the use of adjectives that modify “blinded”: single-, double-, triple-, observer-, personnel-, rater-, observer-, fully or partially blinded, or any other qualifier that would make “blinded” seem more specific than it is. A trial would be described as either blinded or unblinded. Using “blinding” as a verb in a sentence would also be helpful. Such use encourages specificity by requiring a noun, usually which groups were blinded: “We blinded caregivers and data assessors” or “caregivers and data assessors were blinded.”

We wholeheartedly endorse the near-universal recommendation that authors report whether or not the trial was blinded [4, 10, 14–16], who was blinded [1–7, 9–13, 15, 16, 19, 20, 22, 30, 31], how they were blinded [2, 4–6, 12, 13, 19, 20, 26, 30, 31], and whether the method of blinding was likely to be successful [28, 32], including the degree of similarity between the experimental and control interventions [31].

Accordingly, our second proposal is to have all trials described as blinded include the details in a standard “Who Knew” table (Table 2). This table has two parts: a required part and a supplemental part. The required part would indicate whether each of the 6 groups most commonly blinded (the person assigning participants to groups, participants, caregivers, data collectors and managers, outcome assessors, and statisticians) *was or was not blinded*, what information they were blinded to, how blinding was implemented, and whether blinding was maintined during the trial. The supplemental part, used when necessary, would present the same data for any other group or individual who was blinded. Individuals with dual responsibilities, such as caregiving and data collecting, would be identified in the same row heading. If blinding was compromised, authors should report the fact in the table and indicate in the text the potential implications that loss of blinding might have for interpreting the results.

**Table 2:** A standard table for reporting the use of blinding in randomized trials of pharmaceutical interventions

Group or individual blinded^a^	Information withheld^b^	Method of blinding^c,d^	Blinding compromised
**Required fields to be completed for all trials described as blinded**			
Person assigning participants to groups	Group assignment	Concealed allocation schedule	No
Participants	Group assignment	Placebo medications; sham surgeries	No
Care providers	Group assignment	Not told of group assignment	No
Data collectors and managers	Group assignment	Not told of group assignment	No
Outcome assessors	Purpose of study; group assignment; participant characteristics	Participants given numerical identifiers	No
Statisticians	Participant and group identities	Participants and groups given numerical identifiers	No
**Supplemental fields for all blinded groups or individuals not mentioned above**			
Trial manager	Not applicable	. . .	. . .
Pharmacists	Not applicable	. . .	. . .
Laboratory technicians	Participant identities	Participants given numerical identifiers	
Outcome adjudicators	Group assignment	Groups given numerical identifiers	Yes [put details in text]
Data monitoring and safety committees	Not applicable	. . .	. . .
Manuscript writers	Not blinded	. . .	. . .

^a^Other groups or individuals in a trial that were capable of being blinded should be listed in the table, and whether or not they were blinded in the study should be indicated. Individuals with dual responsibilities, such as caregiving and data collecting, should be identified by combining the entries in the same row heading

^b^Although group assignment is the information most commonly withheld in a blinded trial, data assessors, such as pathologists and radiologists, are often blinded to the purpose of the trial, group assignment, and the demographic and clinical characteristics of participants whose biopsy samples or images they are interpreting

^c^In many cases, authors should determine before the trial begins whether the method of blinding had a reasonable chance of being effective, including establishing the similarity between active and placebo preparations and the bioequivalent availability for two or more active drugs [33]. Testing the effectiveness of blinding after the trial has ended is uninformative because the results cannot be separated from pre-trial expectations of the success of the intervention [32]

^d^If blinding has been compromised, authors should report the fact and indicate the potential implications the loss of blinding might have for interpreting the results

## Conclusions

“Blinding” as a concept to reduce bias has been used for more than 200 years [34], and “double-blind” as a term has been used in clinical trials for 70 years [35]. Even with the substantial support in the literature for abandoning its use, finding a simple, acceptable replacement seems unlikely. Instead, eliminating the use of adjectives that impart a false specificity to the term would reduce misinterpretations, and recommending that authors report who was blinded to what and how in a standard table would require them to be more specific about which groups and individuals were blinded.

**Thomas A. Lang, MA**

Principal, Tom Lang Communications and Training International

Adjunct Instructor, Medical Writing and Editing Program, University of Chicago Professional Education

Senior Editor, West China Hospital/Sichuan Medical School, Chengdu, China

**Donna F. Stroup, PhD, MSc**

Principal, Data for Solutions, Inc.

Adjunct Instructor, Medical Writing and Editing Program, University of Chicago Professional Education

**Co-signers** (in alphabetical order):

**Matthias Egger, MD, MSc, FFPH**: Professor of Epidemiology and Public Health and former Director, Institute of Social and Preventive Medicine, University of Bern, and President, National Research Council, Swiss National Science Foundation. Former co-editor, *International Journal of Epidemiology*

**Forough Farrokhyar, MPhil, PhD**: Professor and Research Director, Department of Surgery, Department of Health, Evidence and Impact, McMaster University

**Robert Fletcher, MD**: Professor Emeritus of Population Medicine, Harvard Medical School; founding Co-Editor, *Journal of General Internal Medicine*; former Co-Editor-in-Chief, *Annals of Internal Medicine*; founding member, Word Association of Medical Editors (WAME); member, International Advisory Board, *The Lancet*

**Suzanne W. Fletcher, MD**: Professor Emerita of Population Medicine, Harvard Medical School; founding Co-Editor, *Journal of General Internal Medicine*; former Co-Editor-in-Chief, *Annals of Internal Medicine*; National Academy of Medicine; former member, American Board of Internal Medicine; founding member, US Preventive Services Task Force

**R Brian Haynes, OC, MD, PhD, FRCPC**: Professor Emeritus of Clinical Epidemiology and Biostatistics; Professor of Medicine, McMaster University; co-founder, Evidence-Based Medicine movement; founder, Health Information Research Unit; founding Editor, *ACP Journal Club*; lead developer of the structured abstract

**Anne Holbrook, MD, PharmD, MSc, FRCPC**: Professor, Department of Medicine, and Director, Division of Clinical Pharmacology & Toxicology, McMaster University; leading Canadian drug policy advisor and research lead for evidence-based therapeutics

**Eileen K Hutton, RM, PhD, DSc (HC)**: Professor Emerita and former Assistant Dean, Faculty of Health Sciences, and former Director of Midwifery, McMaster University; Professor of Midwifery Science, Vrije University, Amsterdam; and Fellow, Canadian Academy of Health Sciences

**Alfonso Iorio, MD, PhD, FRCPC**: Professor, Department of Health Research Methods, Evidence and Impact; Bayer Chair for Clinical Epidemiology Research and Bleeding Disorders; Chief, Health Information Research Unit and Hamilton-Niagara Hemophilia Program, McMaster University

**Richard L. Kravitz, MD, MSPH**: Professor, Internal Medicine; Former Director, Center for Health Services Research in Primary Care, University of California, Davis; former co-Editor-in-Chief, *Journal of General Internal Medicine*; Director, UC Center Sacramento, a program providing leadership training in politics and relevant evidence for policymakers

**José Florencio F. Lapeña Jr., MA, MD, FPCS**: Professor of Otolaryngology; former Vice-Chancellor, University of the Philippines; Editor-in-Chief, *Philippine Journal of Otolaryngology Head and Neck Surgery*; Charter President, Philippine Association of Medical Journal Editors; Past President, Asia Pacific Association of Medical Journal Editors (APAME); Secretary and Past Director, World Association of Medical Editors (WAME)

**Maria del Carmen Ruiz-Alcocer, MD**: Senior Medical Editor, Intersistemas Publishers; Former President, Mexican Association of Biomedical Journal Editors (AMERBAC); Past Director, World Association of Medical Editors (WAME); member, European Association of Science Editors (EASE)

**Roberta Scherer, PhD**: Senior Scientist, Clinical Trials and Evidence Synthesis, Johns Hopkins Bloomberg School of Public Health; former Associate Director, USA Cochrane Center; Adjunct Assistant Professor, Epidemiology & Public Health, University of Maryland School of Medicine

**Christopher H. Schmid, PhD**: Professor and Chair of Biostatistics and founding member and former Co-Director of the Center for Evidence Synthesis in Health in the Brown University School of Public Health; founding Co-Editor of *Research Synthesis Methods*; helped develop Institute of Medicine national standards for systematic reviews

**Thomas A. Trikalinos, MD**: Associate Professor of Health Services, Policy, and Practice; Director, Center for Evidence Synthesis in Health, School of Public Health, Brown University

**Junmin Zhang, MD, PhD**: Professor and Managing Director, *Journal of Capital Medical University*, *Medical Education Management*, *Journal of Translational Neuroscience*, Capital Medical University, Beijing, China

## Data Availability

Not applicable
